# The Prevalence and Clinical Correlates of an Auscultatory Gap in Systemic Sclerosis Patients

**DOI:** 10.1155/2012/590845

**Published:** 2012-02-16

**Authors:** Tracy M. Frech, Jason Penrod, Michael J. Battistone, Allen D. Sawitzke, Barry M. Stults

**Affiliations:** ^1^Division of Rheumatology, Department of Internal Medicine, University of Utah, 4B200 SOM, 30 North 1900 East, Salt Lake City, UT 84132, USA; ^2^Department of Internal Medicine, University of Utah, Salt Lake City, UT 84132, USA; ^3^Department of Medicine, Salt Lake City Veterans Affairs Medical Center and the University of Utah, 50 North Medical Drive, Salt Lake City, UT 84132, USA

## Abstract

*Introduction*. Accurate blood pressure (BP) measurement is essential to the diagnosis and management of hypertension in patients with systemic sclerosis (SSc) to help prevent renal and cardiovascular complications. The presence of an auscultatory gap during manual BP measurement—the temporary disappearance of the Korotkoff sounds during cuff deflation—leads to a potentially important underestimate of systolic BP if undetected. *Objectives*. Since the presence of an auscultatory gap is frequently associated with increased vascular stiffness, we investigated its presence and correlates in 50 consecutive SSc patients. *Methods*. For each patient, BP was measured sequentially using three different approaches performed in the same order. *Results*. Sixteen of 50 patients (32%) had an auscultatory gap which if undetected would have resulted in clinically important underestimates of systolic BP in 4 patients. The presence of an auscultatory gap was statistically associated with the presence of antibodies to RNA polymerase III (*P<0.0068*) and SSc diagnosis type (*P<0.01*). *Conclusions*. Our study demonstrates that auscultatory gaps are relatively common in SSc and correlate with markers for SSc vasculopathy. If undetected auscultatory gaps may result in clinically important underestimation of BP. Thus, electronic oscillometric BP may be preferred in SSc patients.

## 1. Introduction

Blood pressure (BP) has most commonly been measured manually using aneroid or mercury sphygmomanometers although electronic oscillometric BP measurement is increasingly used in many office settings [1, 2]. Accurate BP measurement is essential to the appropriate diagnosis and management of hypertension in patients with systemic sclerosis (SSc) to help prevent both renal and cardiovascular complications. Current BP monitoring guidelines in SSc are based on expert opinion. These guidelines have the goal of preventing not only cardiovascular complications, but also scleroderma renal crisis (SRC), which is characterized by increased vascular permeability, activation of the coagulation cascade, and increased renin secretion and which may lead to malignant hypertension, acute renal failure, and death. While most SRC patients have frank hypertension with BP ≥140/90 mmHg, a subset has normotensive SRC heralded by a gradual rise in BP within the prehypertensive range of 120–139/80–89 mmHg [3, 4]. Whether performed manually or electronically, accurate measurement of BP in both office and home settings is of paramount importance to management of SSc [2].

One particular error in manual BP measurement, the failure to detect the presence of an auscultatory gap, results in a falsely low estimate of systolic BP [1, 2, 5]. With an auscultatory gap, the Korotkoff sounds temporarily become inaudible between phase 2 and phase 3 for a few to more than 20 mmHg prior to their reappearance. If a manual BP measurement is performed by auscultation alone, the auscultatory gap may not be detected, and the true systolic BP may be seriously underestimated. To avoid missing an auscultatory gap, the radial artery should be palpated while the cuff pressure is rapidly increased to a level of 30 mmHg above the disappearance of the pulse, followed by auscultation for the Korotkoff sounds during slow deflation of cuff pressure at 2-3 mmHg/second [2]. Inadequately trained medical personnel frequently do not use this technique and may not detect important auscultatory gaps. In one study, 21% of patients with hypertension in a primary care clinic had an auscultatory gap on manual BP measurement [5]. The auscultatory gap does not interfere with BP measurements with electronic oscillometric equipment which measures mean arterial BP with subsequent automatic calculation of estimated systolic and diastolic BP.

The mechanism for an auscultatory gap is uncertain but may in part relate to increased stiffness of the arterial wall [5]. Patients with connective tissue disease may have increased arterial stiffness [6] and therefore may be at increased risk to have an auscultatory gap which if undetected could result in serious underestimation of systolic BP [6]. The presence of an undetected auscultatory gap would be of special concern to patients with impending SRC and could lead to a dangerous delay in their management.

We designed a study to determine the prevalence and clinical correlates of auscultatory gaps in patients with SSc seen at the University of Utah SSc center.

## 2. Materials and Methods

 The study was accepted by the Institutional Review Board at the University of Utah (IRB number 00038705). Inclusion criteria included adult patients (≥18 years) with a diagnosis of SSc as accepted by the American College of Rheumatology [7]. Fifty consecutive SSc patients from the University of Utah SSc Center were recruited and consented at the time of a routine clinic visit between January 2011 and September 2011. For each patient, BP was measured sequentially using three different approaches performed in the same order. Prior to the measurements, each patient emptied their bladder and rested for five minutes seated in a chair with back supported, legs uncrossed, and with their left arm supported with the cuff at midsternal level. Cuff size was properly selected according to midarm circumference with bladder center directly over the brachial artery on a bared arm [2]. No talking was permitted by the patient or examiner during the measurements.

First, the same clinic medical assistant performed a single manual auscultatory measurement using a calibrated aneroid manometer and a high-quality Littmann, Cardiology III stethoscope with short tubing. Without palpating the radial artery, the cuff was inflated to 160–180 mmHg and then deflated at a rate of 2-3 mmHg/second. The systolic BP was noted as the first of two discrete, tapping Korotkoff sounds and the diastolic BP as the disappearance of the Korotkoff sounds.

After a period of five minutes, the BP measurement was repeated by the medical assistant using the Omron HEM-907 device, a validated electronic oscillometric device, which automatically performs three sequential BP measurements at one-minute intervals and averages them. Following another five-minute interval, a single manual BP measurement was performed by the author (T.M. Frech) using the same aneroid manometer and stethoscope as the medical assistant; TF was blinded to the results of the two prior BP measurement approaches. TF palpated the radial artery, inflated the cuff 30 mmHg beyond the disappearance of the radial pulse, and then deflated the cuff at a rate of 2 mmHg/second. The systolic BP was again noted as the first of two discrete, tapping Korotkoff sounds. The presence and magnitude of any auscultatory gap were noted and recorded.

In order to assess for interobserver agreement, another author (J. Penord) assessed ten of the SSc patients. JP was blinded to the results of TF. TF and JP also assessed twelve consecutive patients presenting to the Internal Medicine clinic to assess a comparison of auscultatory gap prevalence.

The presence of the following clinical risk factors for vascular stiffness for SSc patients was noted: age, BMI, presence of proteinuria (trace or greater on urine dipstick), estimated glomerular filtration rate (MDRD formula), tobacco use, and diabetes mellitus. Additionally, risk factors for SRC and evidence of organ damage were documented, including the presence of RNA polymerase III antibodies, prednisone use, pulmonary arterial hypertension (PAH), and interstitial lung disease (ILD). Medications that can affect BP, including current use of nonsteroidal antiinflammatory drugs (NSAIDs), anti-hypertensive agents, and immunosuppressive medications were also recorded.

Nonparametric analysis using the Kruskal-Wallis test was used to look for statistical relationships between categorical data such as the presence of an auscultatory gap and possible arterial stiffness variables: age, diabetes mellitus, tobacco use, and BP medication use. Additionally, potential markers for more severe SSc vasculopathy-immunosuppressant use, nonsteroidal anti-inflammatory (NSAID) use, RNA polymerase III antibody, pulmonary arterial hypertension (PAH), and interstitial lung disease (ILD), were examined for a statistical relationship with the presence of an auscultatory gap. Correction using the Bonferroni method was used to adjust the *P* values for the multiple comparisons performed.

Analyses of variance (ANOVA) were used to examine the relationship of modified Rodnan skin score (mRSS) as a continuous variable with the same arterial stiffness variables. These analyses were performed using R [8].

Intraobserver agreement was used to calculate the number of patients needed for assessment of prevalence in the Internal Medicine Clinic and SSc clinic.

## 3. Results

 In our SSc study population, 92% of subjects were female, 87% were white, and 13% were Hispanic. The age range was 27 to 80 years. The mRSS ranged from 4 to 28, with 85% of patients being in the limited cutaneous SSc subset. There was evidence of end-organ damage due to SSc (PAH and/or ILD) in 65% of patients. In this study 89% of patients were taking one or more antihypertensive agents, most commonly a calcium channel blocker used to treat their Raynaud's phenomenon. An auscultatory gap was heard in 16 patients for a prevalence of 32% in this study population. The mean auscultatory gap in these 16 patients was 6.1 mmHg and ranged from 4 mmHg to 12 mmHg. In two patients the recognition of this gap reclassified them into the hypertensive range (SBP ≥ 140 mmHg). One of these two patients subsequently required hospitalization for scleroderma renal crisis. In two other patients, the auscultatory gap reclassified the patients into the upper portion of the prehypertensive range (systolic BP of 130–139 mmHg). Thus, four patients would have required medication adjustment based on the more accurate recognition of their true BP.

As presented in Table 1, the presence of an auscultatory gap was statistically associated with the presence of antibodies to RNA polymerase III antibody (*P* < 0.0068) and diagnosis type, as categorized by limited or diffuse cutaneous disease (*P* < 0.01). Other potential arterial stiffness or SSc vasculopathy variables—age, tobacco use, diabetes mellitus, BP medication use, NSAID use, immunosuppression use, PAH, and ILD—were not associated with an auscultatory gap. No patients had proteinuria. 

**Figure 1:**
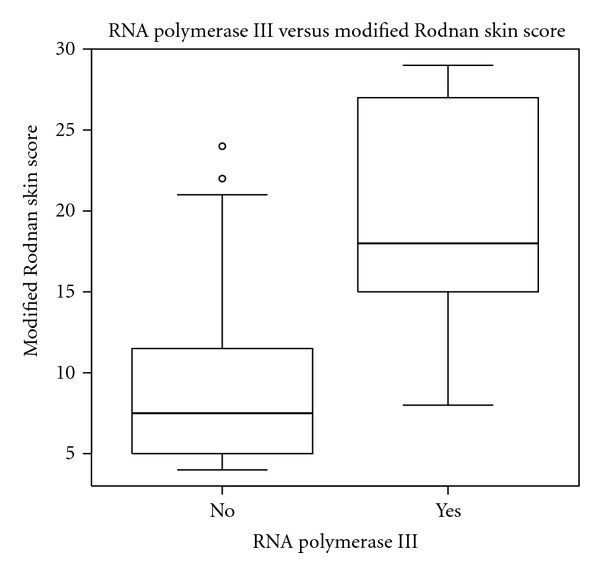


When ANOVA was used to evaluate the relationship of arterial stiffness variables to skin thickness (mRSS), we found age (*P* < 0.02), ILD (*P* < 0.0018), presence of a RNA polymerase III antibody (*P* < 0.00016), and diagnosis type (*P* < 0.000003) were significantly associated. This suggests that looking for an auscultatory gap may be particularly important in older SSc patients with increasing skin score or where RNA polymerase III antibody is present (Figure 1).

When the presence of an auscultatory gap was assessed in 10 of the SSc patients by a second physician, the inter-observer agreement was 0.8. The gap was 4–6 mmHg in the patients in which there was discordance between investigators. Additionally, when the presence of an auscultatory gap was assessed in twelve internal medicine patients, it was found 10–25% of the time. The clinical correlates of auscultatory gap in the Internal Medicine patients were not recorded and are a limitation of this study. The interobserver agreement was 0.83. The gap was 4 mmHg in the patients in which there was discordance between investigators.

## 4. Conclusions

We measured systolic BP by palpation of the radial pulse and then by auscultation at the brachial artery and detected an auscultatory gap in 16 out of 50 consecutively examined SSc patients (32%) in our clinic. In four of the sixteen patients, the magnitude of the auscultatory gap would have resulted in a clinically important underestimate of systolic BP had it remained undetected. Detection of the auscultatory gap reclassified two patients as hypertensive with systolic BPs of 144 mmHg and 148 mmHg, and two other patients were reclassified into the upper portion of the prehypertensive range with systolic BPs of 132 mmHg and 136 mmHg (Table 2). Failure to detect these auscultatory gaps could have seriously underestimated the risk of these patients to develop SRC [9]. Unless health care personnel who measure BP manually in SSc patients are trained to measure systolic BP first by palpation and then by auscultation, clinically important auscultatory gaps may not be detected and systolic BP may be seriously underestimated in a significant minority of SSc patients. Since training medical personnel to accurately measure manual BP is labor intensive and not always successful, we would recommend that BP in SSc patients should be routinely measured using validated electronic oscillometric equipment since estimates of systolic BP are not affected by the presence of an auscultatory gap.

The presence of an auscultatory gap in our SSc patients was statistically associated with the presence of RNA polymerase III antibody, a known marker for increased risk of SRC. It has previously been postulated that the auscultatory gap is a marker of increased vascular stiffness [5]. If our findings are confirmed in another, larger population of SSc patients, the auscultatory gap could be evaluated as a potential readily available clinical marker of risk for SRC.

Skin thickening as measured by the mRSS was correlated to other possible factors that may contribute to arterial stiffness in this SSc population including age, ILD, presence of an RNA polymerase III antibody, and diagnosis type. This suggests that severity of vasculopathy and fibrosis may be a second important reason to detect an auscultatory gap in this patient population. Additionally, if an auscultatory gap is found, a more extensive look for internal organ disease may be warranted.

Our study has some limitations. It is a small study population, and some of the arterial stiffness and SSc vasculopathy variables studied did not have adequate power to exclude their association. For example, only three tobacco users and eight patients with diabetes mellitus were included in the study. Additionally, the use of BP medication at low doses for Raynaud's phenomenon rather than for BP control challenges our use of BP medication as an arterial stiffness variable. Our study population is primarily Caucasian; thus our data may not be able to be generalized to other races and ethnicities.

Nonetheless, our study provides important information on measurement of BP in SSc. An auscultatory gap appears to be common occurring in up to 32% of SSc patients, and failure to detect it may result in clinically important underestimation of systolic BP and missed opportunities to intervene early in hypertensive patients. Future studies should validate whether the auscultatory gap in SSc patients can predict associated internal organ involvement.

## Figures and Tables

**Table 1:** Auscultatory gap association with possible arterial stiffness and vasculopathy variables in SSc.

Arterial stiffness variable	Kruskal-Wallis chi-squared	*P* value
Diagnosis type	8.37	0.01*
Age	26.43	0.43
Tobacco use	0.12	0.73
Diabetes mellitus	0.06	0.81
BP medication	16.32	0.43
NSAID use	0.06	0.81
Immunosuppression	3.00	0.56
RNA polymerase III	7.31	0.01*
PAH	3.43	0.33
ILD	4.29	0.12

SSc: systemic sclerosis; BP: blood pressure; NSAID: nonsteroidal anti-inflammatory use; PAH: pulmonary arterial hypertension; ILD: interstitial lung disease; *P < 0.05.

**Table 2:** Manual blood pressure in SSc patients with auscultatory gap.

Patient	Manual BP without radial pulse occlusion (MA)	Manual BP after pause (TF)	Manual BP with radial pulse palpation (TF)	Auscultatory Gap
1	120/62	118/68	126/62	8
2	130/68	132/72	144/74	12**
3	110/60	112/60	118/62	6
4	104/64	102/68	110/70	8
5	136/64	138/60	148/62	10**
6	114/60	110/54	116/62	6
7	128/80	128/78	136/76	8*
8	126/68	122/68	132/70	10*
9	110/70	104/70	112/64	8
10	110/60	108/64	114/72	6

SSc: systemic sclerosis; BP; blood pressure; MA: medical assistant; TF: Tracy Frech; *change to prehypertensive; **change to hypertensive.
